# “Coffee plus Honey” versus “topical steroid” in the treatment of Chemotherapy-induced Oral Mucositis: a randomised controlled trial

**DOI:** 10.1186/1472-6882-14-293

**Published:** 2014-08-08

**Authors:** Mohammad Ali Raeessi, Neda Raeessi, Yunes Panahi, Homa Gharaie, Seyyed Masoud Davoudi, Alireza Saadat, Ali Akbar Karimi Zarchi, Fereshteh Raeessi, Seyyed Mostafa Ahmadi, Hamidreza Jalalian

**Affiliations:** Department of Otolaryngology, Baqiyatallah University of Medical Sciences, Tehran, Iran; Chemical Injuries Research Center, Baqiyatallah University of Medical Sciences, Tehran, Iran; Natural Medicines Office in Deputy of Food and Drug, Ministry of Health and Medical Educations, Tehran, Iran; Department of Dermatology, Baqiyatallah University of Medical Sciences, Tehran, Iran; Department of Hematology and Oncology, Baqiyatallah University of Medical Sciences, Tehran, Iran; Department of Epidemiology and Biostatics, Faculty of Health, Baqiyatallah University of Medical Sciences, Tehran, Iran; Research Branch, Islamic Azad University of Pharmaceutical Science, Tehran, Iran; Research Center of Baqiyatallah University of Medical Sciences, Tehran, Iran

**Keywords:** Oral mucositis, Cancer chemotherapy, Honey, Coffee, Topical steroid

## Abstract

**Background:**

Oral mucositis is one of the common complications of cancer chemotherapy and about 40% of the patients who take chemotherapy protocols, experience this irritating problem. The purpose of this study was to draw comparison between the therapeutic effects of our treatment modalities (topical steroid, honey, honey plus coffee) in patients suffering from oral mucositis.

**Methods:**

This was a double blinded randomised clinical trial of a total of 75 eligible adult participants which they randomly fell into three treatment groups. For all the participants a syrup-like solution was prepared. Each 600 grams of the product consisted of “20 eight-mg Betamethasone solution ampoules” in the Steroid (S) group, “300 grams of honey plus 20 grams of instant coffee” in the Honey plus Coffee (HC) group, and “300 grams of honey” for the Honey (H) group. The participants were told to sip 10 ml of the prescribed product, and then swallow it every three hours for one week. Severity of lesions was clinically evaluated before the treatment and also one week after the initiation of the intervention. This study adhered to the principles of the Declaration of Helsinki and guidelines of Good Clinical Practice.

**Results:**

This study showed that all three treatment regimens reduce the severity of lesions. The best reduction in severity was achieved in HC group. H group and S group took the second and third places. In other words, honey plus coffee regimen was the most effective modality for the treatment of oral mucositis.

**Conclusion:**

Oral mucositis can be successfully treated by a combination of honey and coffee as an alternative medicine in a short time. Further investigations are warranted in this field.

**Trial registration:**

Iranian Registry of Clinical Trials IRCT: 201104074737N3, (9 May 2011).

## Background

Approximately 57 million deaths occur every year in the world which cancers are responsible for 27% of them [1, 2]. Increased life expectancy, environmental alterations, the changes in lifestyle, potential precise diagnosis, longer life spans and smoking are some of the mentioned causes for increased cancer rates [3, 4].

Cancers are mostly treated with one or more cytotoxic anti-neoplastic agents (chemotherapeutic drugs) as part of a standardised regimen [5]. Anti-neoplastic or cytotoxic drugs trigger all cells with rapid replication cycle. Thereby not only they kill cancer cells, but also hair follicles, bone marrow cells and basal epithelial cells of digestive tract are implicated. Therefore the patients who receive chemotherapy drugs may suffer from mucositis in oral cavity, oropharynx and digestive system [6]. Oral mucositis is one of the common irritating complications of cancer treatment [7, 8]. At least 30-40% of the patients who take chemotherapy drugs, experience some degrees of this oral problem which starts five to ten days after the initiation of the treatment regimen [6].

Oral mucositis is characterised by inflammation, erythema and ulcerations of the mucous membrane of the oral cavity. This mucositis cusses a lot of pain which interferes with normal feeding and may lead to secondary complications such as dehydration, parageusia and malnutrition. In myelosuppressed cancer patients, the infected mucous of the oral cavity can also be a source of systemic infection and septicemia [9]. Undoubtedly, its effective therapy can substantially reduce the oral complications and the risk of oral and systemic infections. One of the common treatments for oral mucositis is topical steroids which is expensive and may have side effects [10–14]. Our suggested treatment is the combination of coffee and honey.

Honey is one of the oldest known natural drugs and since ancient times has been regarded as a health giving substance [15]. Its medical use is recorded from around 3000 B.C. onward and is addressed as a curative substance in the holy Bible. Honey has been highly valued in the Middle East region and it was mentioned as a curative material for human illnesses more than 1400 years ago in the Holy Quran. In traditional medicine, honey has been used for treatment of the signs and symptoms of Upper Respiratory Tract Infections (URTIs), especially coughing [16–20]. The World Health Organization (WHO) has cited that honey demulcents may soothe the throat and therefore it can be a potential treatment for cough and other URTI symptoms [17].

Honey bees produce honey from flower nectar [21, 22].Since honey is not a generic product and its ingredients and their relative amounts depend on the flora of the area from which honey bees collect pollen, it is difficult to determine the composition of honey [23]. It has been reported that honey contains more than 200 substances such as sugars, proteins, minerals, some vitamins, organic acids and antioxidants (phenolic compounds, enzymes, flavonoids, amino acids, carotenoid-like substances and other phytochemicals) [22, 24–26]. According to some studies, honey by its antioxidants can increase cytokine release and has antimicrobial effects [27–29]. Moreover it can prevent cellular oxidative damage which leads to aging, diseases and death [22].

In recent decades, honey has been rediscovered for the maintenance of health in several diseases, burns, injuries, and wounds [17, 30]. It has many medicinal properties including its wound healing capacity [31]. Moreover, honey reduces inflammation and edema, stimulates epithelialisation and tissue regeneration and thus improves granulation and debridement [16, 32, 33] which in turn accelerates tissue repair and leads to wound healing [16, 20, 32–36]. Owing to its therapeutic properties in the treatment of gingivitis and periodontal disorders, honey is also recommended for dental hygiene [37, 38]. Honey by its sweet substances stimulates saliva secretion and also the secretion of mucus in the airways [16, 32, 39]. Previously, some studies have proved promising effects of honey on the cancer treatment-induced oral mucositis [9, 40–42].

On the other hand, Caffeine is a natural alkaloid in coffee, tea, cola drinks, and cocoa [43]. Bronchodilators such as methylxanthines (theophylline and caffeine) stimulate breathing and have been used to prevent apnea [43, 44]. Caffeine is also hypoalgesic and has anti-oxidant and anti-inflammatory effects [43–45]. It is the world’s most commonly used psychoactive substance which stimulates the central nervous system [46]. Caffeine improves psychomotor performance and increases self-reported alertness and decreases self-reported weariness and sleepiness. Coffee per se or its specific compounds contain antioxidant properties and have protective effects against oxidative DNA damage, liver lesions (hepatotoxicant induced fibrosis) and tissue damage [47]. Coffee consumption also attenuates liver induced insulin resistance [48].

To the best of our knowledge, there have been no previous reports about the combination of honey and coffee as a treatment for oral mucositis. While assessing the effect of “honey plus coffee” on the persistence post-infectious cough in our three previous studies [49–51], we noticed the rapid healing impact of this treatment modality on the lesions in hypopharynx mucosal membrane. Thus we decided to design a new trial to evaluate the effect of this regimen on oral mucositis after cancer chemotherapy. Considering the great number of people around the world suffering from oral mucositis, the aim of this study was to evaluate the therapeutic effects of coffee plus honey and compare them with those of topical steroids in the treatment of patients with oral mucositis after cancer chemotherapy.

## Methods

### Design and setting

We have conducted a double-blind randomised clinical trial of 75 adult participants (39 women) presenting oral mucositis after chemotherapy during a period of three years at Baqiyatallah University Hospital, Tehran, Iran from 2011 to 2013.

### Participants

Assuming alpha and beta errors of 5% and 20% respectively, and considering the results significant if the severity of disease changed more than 25% of the mean, we calculated the sample size by its formula: 21 persons for each treatment group. The patients were registered in our clinic after completion of a check list of their personal characteristics including sex, age, weight, education, occupation, and the presence of any other systemic diseases. All the participants provided a comprehensive history and underwent a physical examination of the mouth and throat for any abnormalities. In addition, routine laboratory tests were carried out. Patients with oral mucositis after chemotherapy, and with the age range between 15 and 80 years, were included in the study. Patients with other systemic disease and/or abnormal routine laboratory tests were excluded.

### Ethical considerations

The volunteer participants were informed about the aim and the potential benefits of the study, the prescribed regimens, the follow-up sessions, and their own duty. We informed them about the potential complications including dyspepsia and insomnia, and how to confront them. Informed consent was taken from them before the enrollment in the study. The data and the files have been kept confidential. This prospective trial and its design were approved by the Ethics Committee of Baqiyatallah University of Medical Sciences. The study adheres to the principles of the Declaration of Helsinki and guidelines of Good Clinical Practice. We have registered this clinical trial in the Iranian Registry of Clinical Trials IRCT: 201104074737N3, (9 May 2011).

### Intervention

We utilised online statistical computing web programming (http://www.graphpad.com) to generate the randomisation schedule. Therefore, all the 75 included participants were randomly distributed into three groups and three regimens of medical syrup-like solution were prepared, as follows:

1. Each 600 g of the first regimen consisted of 20 ampoules of Betamethasone (each contained eight mg of Betamethasone solution), given to every member of the Steroid (S) group.
2. Each 600 g of the second regimen consisted of 300 g of honey and 20 g of original instant coffee given to every member of the Honey plus Coffee (HC) group.
3. Each 600 g of the third regimen contained only 300 g of honey as a supportive treatment given to every member of the Honey (H) group.

All the products were in same colour, shape, and taste (by adding enough coffee essence, edible brown colour and artificial honey flavour) and also they had similar packages. The amounts of the ingredients were determined in accordance with usual daily usage. The ingredients were gently mixed and homogenised. We obtained the natural honey from the Zagros Mountains in the west of Iran and also used original instant coffee by Nestle Spain Ltd. The study products should be stored at room temperature of about 22–26°C.

The samples were produced by our pharmacists. We determined the sample sizes using a randomised program and then encoded the samples confidentially and distributed them randomly between the participants. The participants were told to sip three teaspoonfuls (about ten mL) of the prescribed product, and then swallow it. They were asked to repeat it every three hours for one week. The participants were unaware of their own regimen and agreed not to use any additional anti-inflammatory agents, even honey or coffee. In addition, the study investigators were unaware of the prescribed regimen and did not participate as patients in the study.

### Main outcome measures

In all participants the severity of their oral mucositis was clinically evaluated before the treatment and also one week after initiation of the intervention and thereby the checklists were completed. Five experts in the field accepted the validity of our designed questionnaire. With regard to reliability, the questionnaire had a 0.87 of Cronbach’s alpha coefficient. Using a visual analogue score, physicians filled in the questionnaires according to patients’ answers and the participants were then categorised on the basis of the WHO Oral Toxicity Scale for grading oral mucositis. This scale combines both objective (mucosal changes such as redness and ulceration) and functional (inability to eat) outcomes into a single score [52]. Thus the severity of illness was graded as: no oral mucositis (0), mild (1+), moderate (2+), and severe (3+) conditions.

### Statistical analysis

The statistical analyses were carried out using SPSS 16.0 software (SPSS Inc. Chicago, IL). Continuous variables were presented as mean and standard deviation (STD) and categorical variables were presented as absolute and relative frequencies. One-way ANOVA, Chi-square test, Wilcoxon signed ranks test, paired t test and post hoc (Tukey) tests were used to compare the groups. All reported p values were based on two-sided hypotheses. The distribution of the baseline characteristics are shown in Table 1.

**Table 1:** **Characteristics of participants in the three groups: count and percentage within treatment groups**

Factors	S group (n = 21)	HC group (n = 21)	H group (n = 20)
**Gender:**			
Male	11 (52.4)	9 (42.9)	10 (50.0)
Female	10 (47.6)	12 (57.1)	10 (50.0)
**Level of schooling:**			
Primary & Diploma	17 (81.0)	20 (95.2)	15 (75.0)
Higher education	4 (19.0)	1 (4.8)	5 (25.0)
**Occupation:**			
Housewife	6 (28.6)	12 (57.1)	6 (30.0)
Employed	2 (9.5)	3 (14.3)	1 (5.0)
Retired	3 (14.3)	3 (14.3)	4 (20.0)
Unemployed	10 (47.6)	3 (13.6)	9 (45.0)

S = Steroid; HC = Honey plus Coffee; H = Honey.

## Results

**Figure 1:**
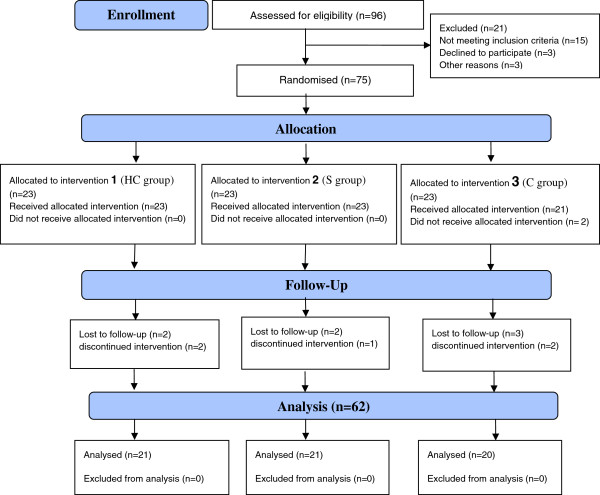
**The flow diagram of the study.**

For each group, the numbers of participants who were randomly enrolled, received intended treatment and were analysed along with the losses and exclusions are depicted in the flow diagram (Figure 1). In this clinical trial the mean (STD) of age and weight were 55.2 (13.2) years, and 77.4 (10.4) kgs respectively. Also the mean (STD) of the severity of illness was 2.57 (0.49) pre-treatment and 0.90 (0.76) post-treatment (Table 2). One-way analysis of variances and Wilcoxon signed rank test, showed that the differences between variables including age and severity of illness before treatment in all three groups were the same and were not statistically significant (p > 0.05, Table 2). The Chi-square test showed that the distribution of variables including sex and education levels were the same in the three groups (p > 0.05).

**Table 2:** **Mean (STD) of differences between treatments and some explanatory variables**

Variables	S group	HC group	H group	P-value
Age (years) (STD)	55.9 (12.7)	54.7 (15.4)	54.9 (11.6)	0.948 (NS)*
Weight (Kgs) (STD)	80.3 (9.2)	73.4 (10.9)	78.4 (10.5)	0.090 (NS)*
**Mean severity of illness (degree) (STD):**				
a- before the treatment	2.52 (0.51)	2.67 (0.48)	2.50 (0.51)	0.517 (NS)*
b- after the treatment	1.43 (0.75)	0.38 (0.50)	0.90 (0.65)	< 0.001(S)**

*NS = Not Significant; **S = Significant; STD = Standard Deviation; S = Steroid; H = Honey; HC = Honey plus Coffee.

The paired t test and post hoc (Tukey) tests showed that the changes in the mean of the severity of illness before and after treatment with every three regimens were statistically significant (p < 0.05). It conveys that generally all three treatment regimens were effective (Table 2), while the mean of changes in severity of illness in the “HC group” was higher than other two groups. To put in a nutshell, the results of this study showed that the honey plus coffee regimen had prominently more curative effect on severity of illness.

## Discussion

Oral mucositis is reported to account for 30–40% of all cases of cancer chemotherapy [6]. The goal of this trial was to evaluate and compare the therapeutic impact of honey plus coffee with topical steroid in patients with oral mucositis after cancer chemotherapy. The results showed that the combination of honey and coffee is a better treatment modality for oral mucositis compared with topical steroid. Eliminating the unpleasant consequences of illness for patients and physicians, this treatment is safe and effective. Honey and coffee are natural edible substances that are agreeable, safe, rather reasonable and easily available. In addition they have proved to be effective in a short period of time. While assessing the effect of “honey plus coffee” on the persistent post-infectious cough in our previous studies [49–51], we noticed the rapid healing impact of this treatment modality on the lesions in hypopharynx mucosal membrane. Thus we decided to design a new trial to evaluate the effect of this regimen on oral mucositis after cancer chemotherapy. In this trial we suggested that the patients take small mouthfuls of the solution, sip and then swallow them. Applying this instruction, our medicinal solution would help healing not only oral cavity lesions, but also any mucosal inflammation in the oropharynx, hypopharynx and esophagus. This is one of the advantages of our suggested treatment regimen.

Although the exact mechanism of action of “honey” is not definitely known, osmolality, acidity, and the production of hydrogen peroxide have been proposed to be the main factors [20]. By reducing prostaglandin synthesis at the site of application, honey lowers plasma prostaglandin concentrations [29]. It also has antioxidant and anti-inflammatory efficacies and increases nitric oxide (NO) in the lesions [29]. It is proposed that, sweet substances per se stimulate the salivation reflex due to their hyperosmolarity. The effects of honey may be related to its hyperosmolarity, anti-inflammatory and antioxidant properties [9, 17, 53–55]. As a result of all these properties, honey can accelerate the repair and healing of mucosal desquamation and thus reduce mucosal irritation [16, 17, 30, 32, 39, 53, 55].

We have chosen the combination of honey and coffee because they are both mentioned in complementary medicine and their mixture is safe and acceptable. It has been reported that honey or coffee separately have treatment impact on some of the respiratory diseases and honey can stimulate mucosal tissue healing in oral mucositis, but to the best of our knowledge, there are no reports in the literature on the combination of these two for oral mucositis. Only our three previous trials showed that “honey plus coffee” regimen is the most effective treatment modality for the Persistent Post-infectious Cough, and also those studies proved that the combination has a greater effect compared with each product separately [49–51].

Yet, it has not been explained how the “combination of honey and coffee” acts, but the impact may be due to the synergistic effect of these two substances. Repairing mucosal desquamation and mucosal irritability, this combination can bring about considerable improvements in mucosal tissue healing.

### Interesting finding

The previous scientific information about caffeine shows it is a psychoactive and hypoalgesic substance, it stimulates breathing and dilates the bronchi and has anti-oxidant/anti-inflammatory effects which protect liver and other tissues [43–47]. Moreover, it reduces liver induced insulin resistance [48]. But with the present study and our other three recent studies we have found a new property for caffeine in combination with honey: “It can speed up wound healing” [49–51].

Further investigations with larger sample sizes are required to make the results more reliable.

## Conclusion

Oral mucositis may be successfully treated by the “combination of honey and coffee” in a short time. Compared with topical steroid, this treatment modality can be used as an alternative medicine in the treatment of oral mucositis. We therefore recommend the use of this effective treatment modality for patients with oral mucositis. Further investigations are warranted.

## Abbreviations

WHO: World Health Organization
URTI: Upper respiratory tract infection
NO: Nitric oxide
STD: Standard deviation.
